# Construction Strategies, Microenvironmental Modelling and Precision-Therapy Applications of Glioma Organoid Models

**DOI:** 10.3390/cancers18162601

**Published:** 2026-08-12

**Authors:** Songming Chen, Wei Zhang, Luohuan Dai, Yubin Kuang, Haodi Yang, Jia Gu, Kang Peng, Nian Jiang, Hongwei Liu, Xuejun Li

**Affiliations:** 1 Department of Neurosurgery, Xiangya Hospital, Central South University, Changsha 410008, China; 2 Hunan International Scientific and Technological Cooperation Base of Brain Tumor Research, Xiangya Hospital, Central South University, Changsha 410008, China; 3 National Clinical Research Center for Geriatric Diseases, Xiangya Hospital, Central South University, Changsha 410008, China; 4 Department of Human Genetics, McGill University, Montreal, QC H3A 0C7, Canada; 5 Department of Radiology, Xiangya Hospital, Central South University, Changsha 410008, China

**Keywords:** glioma, glioblastoma, organoid, tumour microenvironment, precision therapy, drug screening, multi-omics, quality control

## Abstract

Gliomas, and glioblastoma in particular, remain difficult to model because molecular heterogeneity, diffuse invasion, blood–brain and blood–tumour barrier effects, immune suppression and repeated therapeutic escape converge in the same disease. Two-dimensional cultures, glioma stem cell (GSC) systems, acute tumour slices and animal models remain indispensable for mechanistic research, pharmacology and in vivo validation. Glioma organoids are complementary research platforms, not components of routine diagnostic or treatment procedures. This review links model construction, microenvironmental validation, treatment perturbation and evidence-graded interpretation. We compare patient-derived glioma organoids, GSC-derived organoids, brain organoid–glioma co-cultures, genetically engineered brain tumour organoids, and vascular-associated, immune-cell-containing and chip-based platforms. We distinguish phenotypic resemblance from physiological fidelity, tumour-intrinsic drug sensitivity from delivery competence, and proof-of-concept activity from demonstrated clinical utility. We also examine temozolomide resistance, radiotherapy, targeted and combination therapy, antiangiogenic treatment, tumour-treating fields, immune-cell therapy, oncolytic viruses, multi-omic quality control and prospective validation. Organoids should not substitute for animal models or clinical trials. Their most defensible role is to provide a patient-derived functional layer between mechanism, regimen ranking and molecular tumour-board interpretation, with claims limited by assay reproducibility, clinically achievable exposure and outcome linkage.

## Simple Summary

Gliomas, especially glioblastoma, are difficult to model because their cells are heterogeneous, infiltrate the brain, interact with neural, vascular, immune and extracellular-matrix compartments, and frequently escape treatment. This review evaluates glioma organoids as experimental research models rather than established diagnostic or treatment procedures. We compare patient-derived, glioma stem cell-derived, brain organoid co-culture, genetically engineered, vascular-associated, immune-cell-containing and chip-based platforms. We explain how to distinguish a visually similar phenotype from validated physiological fidelity, how to separate tumour-cell drug sensitivity from drug delivery, and how to interpret organoid results according to the strength of the supporting evidence. A question-driven framework, practical quality-control criteria and transparent reporting standards are proposed. Glioma organoids can complement two-dimensional cultures, tumour slices, animal models and clinical studies, but they cannot replace them.

## 1. Introduction

Glioma modelling has both practical and biological urgency. Molecular diagnosis can shape trial eligibility and treatment discussion within days after surgery, whereas many in vivo systems cannot return functional data within that interval. Organoids occupy an experimental space between immediate histopathology and slower animal validation. They are research models rather than diagnostic procedures: when used with restraint, they can test whether a sampled tumour region remains sensitive to a plausible regimen, whether the invasive edge differs from the core, or whether resistant cells expand after treatment withdrawal.

Gliomas are now understood less as purely morphological entities and more as molecularly defined central nervous system tumours. Integrated diagnosis has made IDH status, 1p/19q codeletion, histone alterations, methylation class and other molecular features central to classification and treatment planning [1]. The clinical outlook nevertheless remains poor. High-grade gliomas, especially glioblastoma (GBM), infiltrate brain tissue, recur after maximal therapy and evolve under radiotherapy, temozolomide (TMZ), targeted agents and immune pressure [2]. The blood–brain barrier (BBB) and blood–tumour barrier (BTB) create uneven drug exposure, while tumour cells move among genetic clones and plastic cell states that facilitate therapeutic escape [3,4].

These features create a technical and translational modelling gap. Two-dimensional cultures are fast and perturbable but lose architecture and much of the parental state; glioma stem cell (GSC)/neurosphere cultures enrich stem-like populations but omit many spatial niches; acute slices preserve local architecture for a short interval; and animal models provide systemic and vascular context but are slower, costlier and affected by species differences [5,6,7,8,9]. Organoids add value only when they preserve a patient- or niche-specific feature that a simpler system cannot provide within the same time and resource constraints.

Glioma organoids are therefore most useful as research platforms for questions that fall between reductionist cell biology and whole-animal or clinical investigation. They can make three-dimensional architecture and controlled functional perturbation experimentally accessible, but their output remains model-dependent evidence rather than a treatment recommendation [10,11,12,13,14]. The field has progressed from patient-derived and engineered organoids to co-culture, vascular-associated, immune-cell-containing, chip-based and outcome-linked platforms; this progression represents diversification of research capability, not a monotonic increase in clinical validity.

This review follows six linked questions. What qualifies as a glioma organoid rather than a generic sphere? Which construction strategy fits each molecular glioma entity and research problem? How can organoids model hypoxia, neural activity, vasculature, immunity and mechanics without confusing culture artefacts with physiological fidelity? How should drug sensitivity be separated from drug delivery? How should therapeutic endpoints and evidence levels be interpreted? Finally, what quality-control, reporting and prospective-validation standards are needed before organoids can contribute to precision neuro-oncology? The central argument is that glioma organoids are patient-relevant experimental systems whose claims must follow the model, the validation layer and the level of clinical evidence.

### Literature Search and Selection

For this review, PubMed/MEDLINE was searched with the ending date set at 31 July 2026 and combinations of the terms “glioma”, “glioblastoma”, “diffuse midline glioma”, “organoid”, “tumoroid”, “spheroid”, “brain organoid”, “microenvironment”, “drug screening”, “radiotherapy”, “immunotherapy”, “blood-brain barrier”, “microfluidic”, “multi-omics” and “precision medicine”. Reference lists of relevant primary studies, protocols and recent reviews were also screened. Peer-reviewed English-language studies were prioritized; preclinical proof-of-concept studies, retrospective outcome-linked studies and prospective clinical studies were distinguished.

## 2. Concepts and Technological Evolution of Glioma Organoids

### 2.1. Definition and Boundaries of Glioma Organoids

Terminology matters because it sets the strength of the claim (Table 1). A spheroid may be suitable for testing whether a compound kills tumour cells during three-dimensional growth, but it cannot by itself support claims about brain-like tissue organization, tumour–host interaction or patient-level therapeutic prediction. For that reason, authors should state the starting material, culture format, fidelity evidence and intended readout before describing a model as a glioma organoid.

An organoid is more than a cell aggregate. It is a self-organizing three-dimensional tissue model generated from stem cells, progenitors or patient-derived tissue under conditions that preserve at least part of tissue architecture, cellular state and function [15,16]. In glioma research, the term needs more precision than spheroid or tumoroid. Spheroids emphasize aggregation, neurospheres enrich neural or glioma stem-like cells, and tumoroids broadly describe tumour-derived three-dimensional growth. Glioma organoids, particularly glioblastoma organoids (GBOs) and patient-derived organoids (PDOs), should show parental tumour fidelity, spatial organization, cell-state diversity and measurable treatment responses.

A practical evaluation framework has four parts: structure, state, function and fidelity. Structure concerns reproducible three-dimensional morphology and spatial domains. State asks whether malignant, stem-like, proliferative and, when relevant, non-malignant or host-related populations are retained. Function includes measurable invasion, drug response, radiotherapy response, immune killing or regrowth after treatment. Fidelity requires correspondence to the matched parental input—ideally the specific sampled fragment as well as the broader surgical specimen—across pathology, driver alterations, copy-number changes, methylation class and omic states [11,17]. Because regional heterogeneity is substantial, “parental tumour fidelity” should never imply that one organoid represents the entire tumour unless multi-region sampling supports that claim.

### 2.2. Scope Across Molecularly Defined Glioma Entities

The evidence base remains dominated by adult IDH-wildtype glioblastoma, and the broader term “glioma organoid” is retained because patient-derived and engineered platforms increasingly address IDH-mutant astrocytoma, oligodendroglioma, diffuse midline glioma and other pediatric high-grade gliomas; however, these entities differ in growth requirements, developmental context, molecular stability and treatment relevance (Table 2). Findings from GBM should therefore not be generalized automatically to all gliomas [18].

Establishment success can itself introduce biological selection. Slow-growing IDH-mutant and oligodendroglial tumours may be difficult to maintain, whereas rapidly proliferating or treatment-adapted clones can be preferentially retained. Culture conditions may also alter IDH-associated metabolism, lineage states, native immune/vascular compartments and regional heterogeneity. Studies should report successful and failed cultures, the sampled tumour region, prior treatment, time in culture and whether fidelity refers to the matched input fragment or to the whole surgical specimen.

### 2.3. Technological Evolution

As models become more elaborate, terminology should become more disciplined rather than looser. “Organoid” should indicate a defined construction method, an explicitly validated level of fidelity and a functional research question. Added complexity does not automatically increase physiological fidelity or clinical usefulness; it creates additional variables that must be measured and controlled.

The evidentiary standard has also shifted. Early three-dimensional systems mainly asked whether glioma cells could grow in a spatially organized form. Current studies need to ask whether the model retains the parental tumour state, reconstructs the relevant niche to a measurable degree and provides information that a two-dimensional assay cannot. In this sense, the field has moved from model demonstration toward model qualification.

**Figure 1:**
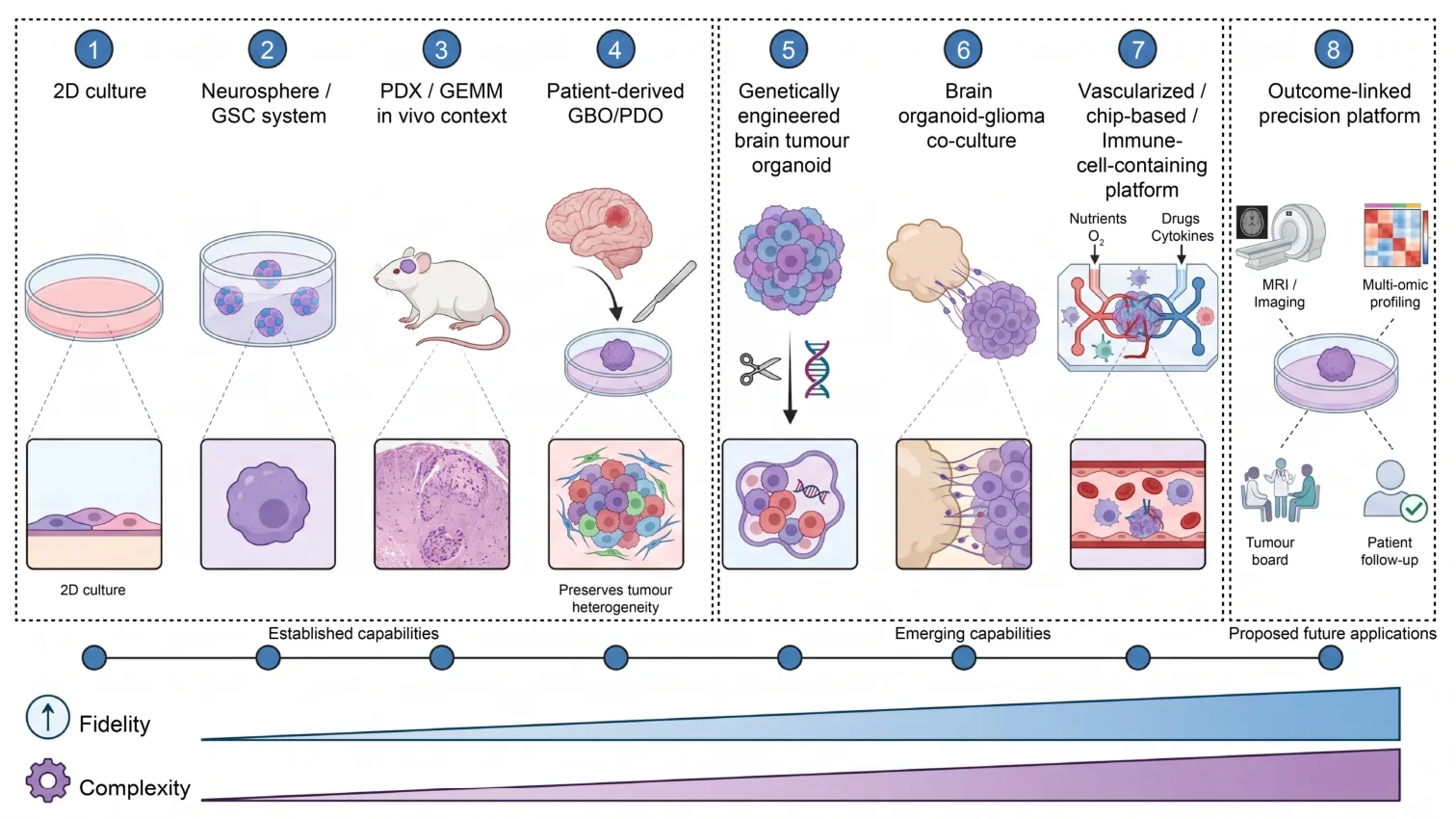
Technological evolution of glioma organoid models. Glioma models have moved from simple two-dimensional and neurosphere systems toward patient-derived, engineered, co-culture, vascularized, immunized, chip-based and outcome-linked platforms, with increasing fidelity and complexity. GSC, glioma stem cell; PDX, patient-derived xenograft; GBO, glioblastoma organoid; PDO, patient-derived organoid. Created with BioRender.com.

Glioma models have diversified from highly expandable systems toward platforms designed for stronger tissue or niche fidelity (Figure 1). Two-dimensional cultures and GSC/neurosphere systems remain valuable for reproducibility and genetic perturbation; acute slices preserve native local architecture for short-term experiments; and patient-derived xenograft (PDX) or genetically engineered animal models provide vascular, immune and systemic context. Patient-derived GBOs demonstrated that surgical tissue can retain histology, genetic alterations and interpatient heterogeneity in three-dimensional culture [10,11,12,13,14]. Engineered brain tumour organoids enabled causal testing of driver cooperation [19,20], brain organoid–glioma co-cultures added a human neural context for invasion [21], and vascular-associated, immune-cell-containing and chip-based platforms introduced controlled barrier, immune and gradient variables [22,23,24,25,26,27,28,29,30,31]. These remain research platforms with different evidentiary strengths rather than a single hierarchy of increasing clinical readiness.

## 3. Construction Strategies and Model Types for Glioma Organoids

**Figure 2:**
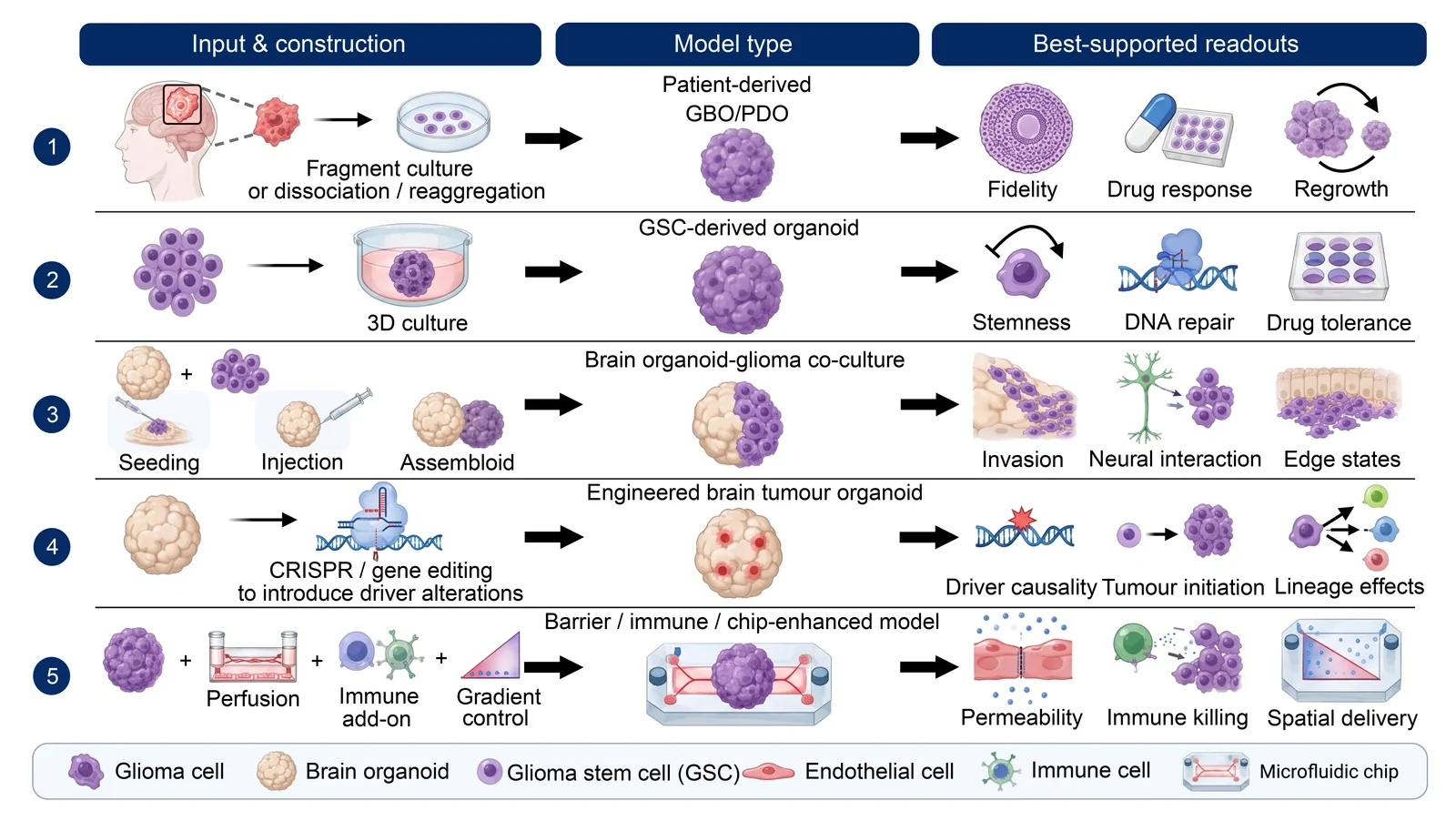
Question-matched construction strategies and best-supported readouts for glioma organoid models. The framework links input and construction to five model categories and their most defensible readouts: patient-derived GBO/PDOs for fidelity, drug response and regrowth; GSC-derived organoids for stemness, DNA repair and drug tolerance; brain organoid–glioma co-cultures for invasion, neural interaction and invasive-edge states; engineered brain tumour organoids for driver causality, tumour initiation and lineage effects; and barrier-, immune- or chip-enhanced models for permeability, immune killing and spatial delivery. Model complexity does not automatically confer physiological fidelity or clinical validity. GSC, glioma stem cell; GBO, glioblastoma organoid; PDO, patient-derived organoid. Created with BioRender.com.

Glioma organoids are a family of question-matched models rather than one technology. Figure 2 links input and construction, resulting model type and best-supported readouts to five research aims: patient-specific response, cell-intrinsic resistance, brain invasion and neural interaction, driver causality, and barrier, immune or spatial-delivery testing. Added complexity does not itself establish physiological fidelity or clinical validity; the platform must fit the inference being made.

### 3.1. Patient-Derived GBO/PDOs

Patient-derived GBO/PDOs are the closest organoid format to a surgical specimen, but their design involves a trade-off. Fragment-based GBOs better preserve local architecture, necrotic or proliferative zones, extracellular matrix and pre-existing cell–cell contacts, whereas dissociated and reaggregated PDOs are easier to standardize by input number and often suit quantitative screening. Protocol choice can itself reshape tumour architecture and identity [11,27,32]. Neither approach is inherently superior; the endpoint and the required fidelity layer should determine the format.

The main caveat is that fidelity can fade without an obvious morphological warning. A culture may continue to grow while minor clones, treatment-resistant or non-proliferative populations, native immune cells or spatially restricted states disappear. Short-term functional assays should therefore be distinguished from long-term biobank expansion. Reports should state the sampled region, viable tumour fraction, culture duration, passage or timepoint, input fragment size or cell number, establishment success and the quality-control (QC) layer used before treatment testing.

The strongest evidence for this format comes from studies showing that GBOs generated from surgical tissue can retain parental histology, cellular composition and genetic alterations, with later protocols standardizing generation, cryopreservation and functional testing [11,32]. Related PDO approaches have also been extended to lower-grade glioma and recurrent disease, widening the clinical contexts in which patient-derived organoids can be used [33,34,35,36,37].

PDO/GBO workflows may be short-term or expandable. Short-term organoids are useful when the goal is to preserve the surgical state and test a focused set of clinically relevant perturbations quickly. Expandable models support repeated experiments, biobanking and cross-batch validation, but prolonged growth can preferentially retain rapidly proliferating clones and lose quiescent, treatment-resistant or microenvironment-dependent populations. Successful cultures should therefore be interpreted together with failed cultures and longitudinal fidelity checks.

Strong PDO/GBO studies therefore rely on layered validation rather than a single marker stain. H&E and immunostaining describe morphology; driver and copy-number testing check molecular fidelity; methylation classification anchors diagnosis; and functional assays link the model to treatment response. Single-cell or spatial omics can add useful resolution, but they should be matched to the claim rather than used decoratively.

The most useful outputs are not limited to viability. Histological similarity, molecular fidelity, baseline growth, treatment response, invasion and regrowth after withdrawal all matter. QC can be stated without a long marker list: histology, key immunomarkers, driver alterations, methylation class and functional response should be reported according to the question. This makes PDO/GBOs well suited to individualized testing and outcome-linked studies, but less suited to proving isolated driver causality on their own.

### 3.2. GSC-Derived Organoids

GSC-derived organoids are valuable when the study needs experimental control. They support matched perturbations, rescue experiments and repeated drug-pressure assays that would be difficult in heterogeneous surgical fragments. Their strength is not complete tumour reconstruction; it is a stable three-dimensional system for testing cell-intrinsic mechanisms.

These models usually begin with patient-derived GSCs, neurospheres or stem-like tumour cells expanded under neural stem cell conditions and then organised in three-dimensional culture. Hubert and colleagues showed that GBM organoids can reproduce hypoxic gradients and cancer stem cell heterogeneity [10]. Their strength is reproducible perturbation of cell-intrinsic stemness, DNA repair and resistance pathways; they do not reconstruct the complete patient tumour ecosystem.

Useful readouts include stemness markers, clonogenicity, invasion distance, migration speed, DNA-damage repair, drug tolerance and cell-state transitions. The advantage is reproducibility and perturbability. The limitation is drift: long-term culture can select clones and shift cell states, so these organoids should not be read as a complete patient tumour ecosystem [38]. They are best used for stemness, invasion and resistance mechanisms, followed by validation in patient-derived systems when translation is the goal.

### 3.3. Brain Organoid–Glioma Co-Cultures

Brain organoid–glioma co-cultures (GLICO model) are particularly suited to investigate tumour–brain interface behaviours, whereby glioma cells can spread over the organoid surface, infiltrate following internal injection, or invade across assembloid boundaries. Importantly, these experimental configurations address distinct biological questions: surface seeding enables visualization of outside-in invasion, direct injection recapitulates tumour growth within a brain-like tissue milieu, and assembloid platforms generate a more consistent tumour–brain interface for comparative analyses. Collectively, the strength of such systems resides in embedding glioma cells within a human three-dimensional neural microenvironment. While GLICO and related protocols introduce GSCs into cerebral organoids via seeding or injection, emerging assembloid technologies facilitate the construction of better-defined tumour–brain interfaces [20,21,39,40,41,42,43,44]. Once functionally validated, these culture formats support live imaging of invasion, assessment of host-dependent tumour phenotypic plasticity, and interrogation of neuron–tumour communication.

Key readouts include invasion distance, invasion area, migration speed, invasive-front cell states, live-cell trajectories and, when the claim involves neural coupling, calcium imaging or electrophysiology. The main limitations are incomplete brain organoid maturation, regional variability, lack of mature vasculature and limited immune content. These models are best used for invasion, host-dependent growth and neuron–tumour communication hypotheses, rather than for full ecosystem reconstruction.

### 3.4. Genetically Engineered Brain Tumour Organoids

Genetically engineered brain tumour organoids are most persuasive when used to ask causal questions. They can show that a defined alteration is sufficient or cooperative in a developmental setting, while patient-derived GBOs can test whether the same pathway remains active after years of tumour evolution and therapy pressure. Used together, the two systems create a stronger chain of inference: engineered organoids nominate mechanisms, and patient-derived models test their relevance in authentic tumour backgrounds.

Rather than preserving a patient tumour as found, engineered organoids introduce defined driver alterations into hESC- or iPSC-derived brain organoids using CRISPR/Cas9, PiggyBac, viral transduction or knock-in/knockout strategies. Bian and colleagues used combinations involving MYC, HRAS, AKT, TP53, NF1 and PTEN to model tumour formation, while Ogawa and colleagues introduced HRasG12V into a TP53 locus to induce a GBM-like phenotype [19,20].

This strategy is useful when the question asks whether a driver event is sufficient, how mutations cooperate, or how developmental stage and regional context affect tumour initiation. Alterations in TP53, PTEN, NF1, EGFR/EGFRvIII, PDGFRA, IDH1 or H3K27M can help model adult, lower-grade or pediatric-like glioma programmes [45,46,47]. Still, engineered models are not stand-alone precision-treatment predictors because they lack authentic patient evolution, therapy selection, immune pressure and mature vascular context. Their best use is tumour origin, driver cooperation and subtype-specific mechanism.

### 3.5. Vascular-Associated, Immune-Cell-Containing and Chip-Based Organoids

For the question-driven framework in Figure 2, vascular-associated, immune-cell-containing and chip-based platforms are grouped as barrier-, immune- or chip-enhanced models; this umbrella label denotes selected added components and does not imply complete reconstruction of the tumour ecosystem. Vascular-associated systems need to report both structure and performance. Endothelial markers, branch length and vessel-like networks are useful structural checks, but functional claims about BBB/BTB behaviour require permeability, transporter expression, barrier resistance, perfusion and spatial drug-distribution measurements. In many studies, “vascular-associated” is more accurate than “fully vascularized”. A submerged organoid can reveal tumour-intrinsic pharmacodynamic sensitivity, but it cannot establish delivery competence across the BBB/BTB or clinically achievable intratumoural exposure.

Immune-cell-containing models require similar restraint. Organoids that transiently retain native immune cells should be distinguished from reconstructed systems in which microglia, monocyte-derived macrophages, T cells, CAR-T cells or CAR-NK cells are added. Autologous and allogeneic systems are not biologically equivalent. Studies should report immune-cell source, HLA compatibility, activation state, tumour-to-effector ratio, medium, exposure duration, retention and viability. These models cannot reproduce systemic immunosuppression, lymphoid-organ interactions, bone-marrow T-cell sequestration, corticosteroid exposure or treatment-related systemic toxicity.

Chip-based models add an engineering layer because they make gradients visible and adjustable. Flow rate, shear stress, oxygen tension and drug exposure can be controlled, which helps studies of drug penetration or hypoxia-driven invasion. The trade-off is lower throughput and greater variation across devices. Chip studies should therefore report enough device and exposure information for replication, while avoiding broad claims from a single geometry.

A useful design principle is to add only the component needed for the question. For a BBB-penetrating nanoparticle, endothelial barriers, perfusion and spatial drug tracing matter more than a broad immune add-on. For CAR-T testing, antigen distribution and immune-cell retention matter more than a sophisticated hypoxia chip. This kind of restraint keeps complex models interpretable.

Vascular-associated, immune-cell-containing and chip-based models add selected ecosystem variables that simpler organoids lack. Vascular modules address perfusion, nutrient supply, hypoxia and BBB/BTB-related delivery; immune modules examine retention, infiltration, killing and antigen escape; and microfluidic systems control flow, shear stress, oxygen and drug gradients [28,48,49,50,51,52,53,54,55,56,57,58,59,60,61,62,63,64]. Added components also increase cost, reduce throughput and create new batch effects. These platforms should supplement PDO/GBOs when barrier function, immunity or gradient control is central, not be treated as superior merely because they are elaborate.

Relevant outputs include perfusion, permeability, tight-junction markers, immune infiltration, cytokine release, killing coverage, antigen loss and spatial drug distribution. The recurring limitation is technical complexity: added components usually increase cost, reduce throughput and create new batch effects. These models should supplement PDO/GBOs when barrier function, immunity or gradient control is central; they should not be treated as superior simply because they are elaborate.

### 3.6. Model Selection

A practical model-selection rule is to separate the inference being sought. Patient-specific response generally starts with fresh or short-term GBO/PDOs. A cell-intrinsic resistance pathway may start with GSC-derived organoids and return to patient-derived material for confirmation. Invasion into brain-like tissue requires co-culture or assembloid designs. Delivery, immunity or hypoxia under flow calls for barrier, immune or chip-based systems. Acute slices are preferable when short-term preservation of the native local microenvironment is essential, whereas orthotopic PDX or genetically engineered animal models are required for systemic pharmacokinetics, whole-organ toxicity and organism-level immune or vascular effects.

Model pairing often gives the stronger design. A PDO/GBO can nominate a patient-specific drug response, a GSC-derived organoid can test whether that response depends on a cell-intrinsic pathway, a co-culture model can examine invasive-edge effects, and a chip model can test delivery. Triangulation of this kind is usually more convincing than reliance on one platform.

No single model occupies the apex of a universal evidentiary hierarchy; robust experimental design typically triangulates complementary platforms: PDOs/GBOs can identify patient-specific treatment responses, GSC-derived organoids enable dissection of cell-intrinsic mechanisms, brain organoid co-cultures facilitate investigation of invasive edge phenotypes, barrier-on-chip systems allow testing of drug delivery, and orthotopic models support evaluation of in vivo drug exposure and systemic effects. Table 3 contrasts these platforms in terms of experimental timeline, throughput, retained biological components, and key biological questions each system cannot adequately address. Organoids warrant their added technical complexity only when they recapitulate patient-specific tissue architecture, spatially resolved residual tumour states, human tumour–brain interfaces, or controllable niche variables inaccessible via simpler assays. By contrast, two-dimensional cultures and GSC models are optimal for high-throughput genetic perturbation, acute tissue slices excel at short-term preservation of native tumour tissue, and PDX or genetically engineered mouse models (GEMMs) are preferred for studying systemic drug delivery, toxicology, and in vivo immune and vascular responses. Accordingly, every study should explicitly define its experimental comparators and clarify the incremental biological insights afforded by organoid models under realistic time and resource limitations.

## 4. Modelling the Tumour Microenvironment with Glioma Organoids

**Figure 3:**
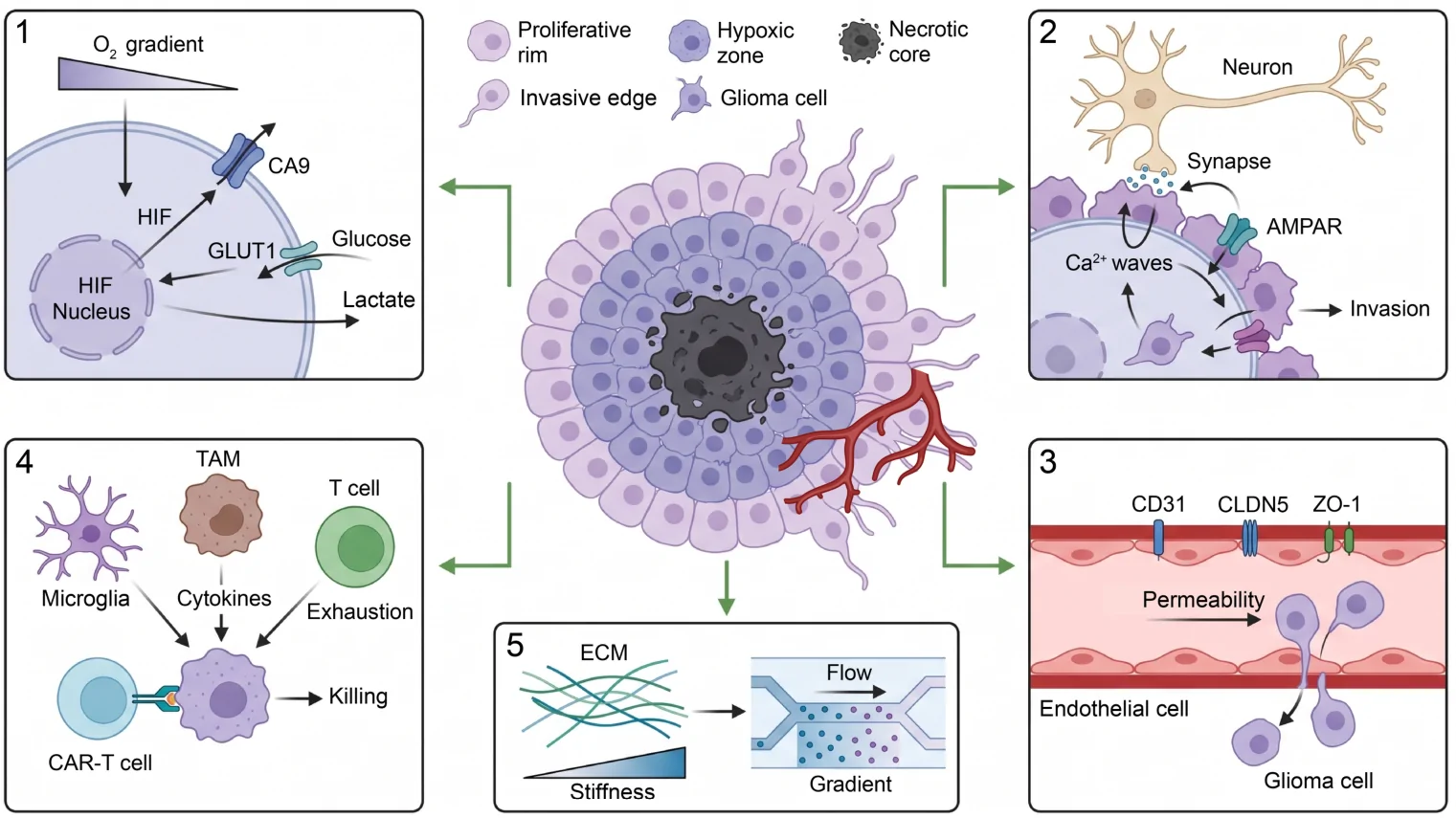
Modelling the glioma microenvironment with organoids. The central organoid contains proliferative, hypoxic, necrotic and invasive zones, while surrounding modules show measurable hypoxia–metabolic gradients, neuron–tumour communication, BBB/BTB-related delivery, immune suppression and ECM or chip-controlled gradients. ECM, extracellular matrix; AMPAR, α-amino-3-hydroxy-5-methyl-4-isoxazolepropionic acid receptor. Created with BioRender.com.

Glioma organoids are useful not simply because they grow in three dimensions, but because selected niche pressures can be reconstructed and measured. Phenotypic resemblance must be separated from physiological fidelity (Figure 3). A CD31-positive structure is not automatically a functional BBB; tumour entry into a brain organoid is not automatically a neuron–tumour synapse; immune-cell addition is not automatically immunosuppression; and a hypoxic or necrotic core may be a size-dependent diffusion artefact rather than mechanistic equivalence to the patient tumour. Structural markers should therefore be paired with functional perturbation, matched-tissue comparison and appropriate artefact controls [23,24,25,28].

### 4.1. Hypoxia, Necrosis and Metabolic Gradients

Bulk response can be misleading in hypoxic organoids. A drug may reduce peripheral proliferation while sparing a hypoxic core, or kill central stressed cells while leaving a migratory rim. Spatial sampling after treatment can reveal whether the residual population reflects a pre-existing niche, a therapy-induced state or non-specific culture stress. Endpoint viability curves alone cannot make that distinction.

Hypoxia should be treated as both a biological feature and a culture-dependent variable. Organoid diameter, cell density, oxygen consumption, medium depth and perfusion all shape oxygen and nutrient gradients. The presence of HIF-1α, CA9, necrosis or a proliferation gradient establishes a hypoxic phenotype, but not by itself mechanistic equivalence to clinical pseudopalisading necrosis, which also reflects abnormal vessels, heterogeneous perfusion, edema, metabolic competition and treatment-related changes.

Biologically meaningful hypoxia modelling should combine pre-specified organoid-size ranges, directly measured oxygen gradients or validated hypoxia probes, spatial metabolic analysis, comparison with matched patient tissue, and controlled perturbation of oxygenation or vascular supply. Where applicable, functional perfusion or permeability should also be measured. A phenotype that changes appropriately when oxygen or flow is manipulated provides stronger evidence than a static marker pattern.

Organoid size and culture time should therefore be treated as experimental variables rather than background details. Studies should report size distributions, sampling timepoints, diffusion distance and the proportion of organoids that develop spontaneous hypoxia or necrosis. Size-matched controls are essential when therapeutic responses may depend on an artefactually hypoxic core.

Useful readouts include HIF-1alpha/HIF-2alpha, CA9, GLUT1, pimonidazole labelling, Ki-67, cleaved caspase-3, necrotic fraction, lactate, glucose uptake and spatial transcriptomic differences between rim and core (Table 4). Hypoxia can support glycolysis, stem-like maintenance, invasion, TMZ tolerance and radioresistance [65,66,67,68]. Central necrosis should not be equated with pathological pseudopalisading necrosis unless histology, oxygen/metabolic measurements, matched-tissue comparison and perturbation data support that claim.

### 4.2. Neuron–Tumour Interaction and Invasive Niches

This distinction matters for therapeutic testing. A compound that reduces invasion through brain-like tissue may not disrupt neuron–tumour synapses, and a drug that modulates neuronal activity may not directly suppress tumour viability. Organoid co-cultures can separate these possibilities if imaging, transcriptional profiling and functional neural assays are aligned. The best studies should define whether they are testing migration, activity-dependent growth, synaptic integration or some combination of these processes.

Glioma is a brain-network disease as well as a tumour-cell disease. Neuronal activity-regulated neuroligin-3 can promote high-grade glioma growth, and glioma cells can receive glutamatergic synaptic input through AMPAR-mediated neuron–tumour synapses [69,70,71,72]. Brain organoid–glioma co-cultures provide a human neural context in which invasion and host interaction can be observed, especially in GLICO and related models [21,39].

Invasion models should quantify invasion distance, area, migration speed, tumour-cell distribution and invasive-edge transcriptional states (Table 4). Functional neural-interaction models need stronger evidence, such as synaptic markers, calcium imaging, electrophysiology and neuronal-activity perturbation. A tumour cell that enters a brain organoid has not necessarily integrated into a neural network. When the question is invasion, imaging and spatial transcriptomics may be sufficient. When the question is neural coupling, electrophysiological or activity-based evidence becomes necessary. That threshold should be clear before interpretation.

### 4.3. Vessels, BBB/BTB and Drug Delivery

This issue is especially relevant for compounds that appear active in molecular screens. Tumour-cell target engagement in a submerged organoid demonstrates pharmacodynamic sensitivity but says little about BBB/BTB penetration, systemic pharmacokinetics, metabolism, toxicity or achievable intratumoural exposure. Barrier and chip designs can test whether delivery limits a specific hypothesis, but only when transporter expression, permeability or resistance, perfusion and spatial drug distribution are functionally demonstrated.

Vascular and barrier features are central to glioma therapy because many drugs fail before they reach infiltrative tumour cells. Vascularized organoids and BBB/BTB chips can model oxygen and nutrient supply, perivascular niches, permeability and trans-barrier transport. Yet vessel-like markers alone do not establish a mature BBB. Structural vessel-like readouts such as CD31, VE-cadherin and branch length should first establish vascular-associated organization [48,49,50,51,52]. Claims about BBB/BTB-like function require stronger evidence, including CLDN5, ZO-1, transporter expression, perfusion status, permeability assays and drug tracing [53,61,62,63,64].

Barrier modelling should be aligned with pharmacology. Tested concentrations should be related, where possible, to clinically achievable unbound plasma or brain exposure rather than arbitrary high doses. For small molecules, permeability, efflux, protein binding and free intracranial concentration are central; for nanoparticles or delivery vehicles, penetration depth, perivascular retention and spatial pharmacodynamic effect are more informative. The terms vessel-like, barrier-associated or BBB/BTB-like should be used unless mature function is directly shown.

For delivery studies, the best models combine barrier readouts with drug mapping (Table 4). A compound or nanoparticle should be traced across the barrier, through the tumour mass and into the invasive edge if the claim concerns therapeutic delivery. Fluorescent tracing, mass-spectrometry imaging or spatial pharmacodynamic markers can help distinguish true delivery failure from intrinsic resistance. This distinction matters because a drug that works in a submerged organoid may fail in the brain simply because it never reaches the relevant cells.

### 4.4. Microglia, Macrophages and Immunosuppression

For immune models, dynamic readouts are usually more informative than static markers. Marker expression can show that immune cells are present, but not whether they infiltrate, become suppressed, kill only antigen-high regions or leave resistant clones. Time-lapse imaging, cytokine profiling, flow cytometry, spatial transcriptomics and post-treatment residual-cell mapping should be interpreted with immune-cell source, HLA compatibility, activation state, tumour-to-effector ratio, medium and duration. Native-immune-retaining, autologous reconstructed and allogeneic systems should be analyzed as distinct platforms.

The GBM immune microenvironment is dominated by myeloid populations and functionally impaired lymphocytes. Microglia and macrophages differ in origin, spatial distribution and tumour education [73,74,75,76,77]. Immune-cell-containing organoids can preserve endogenous immune cells or add microglia, monocyte-derived macrophages, T cells, CAR-T cells or CAR-NK cells. Source, timing, ratio and retention should be stated because each variable changes the result.

Immune-cell-containing organoids should also distinguish autologous from allogeneic designs. Autologous systems preserve patient-specific tumour-immune compatibility, but they are difficult to establish and often limited by available cell numbers. Allogeneic or engineered effector systems are easier to standardize, yet they may introduce mismatch biology. A caveat of this sort prevents the model from being read too broadly.

Useful readouts include immune-cell retention, infiltration depth, phagocytosis, antigen presentation, cytokine release, exhaustion markers, killing area, antigen loss and residual-cell state (Table 4). These data should be paired with flow cytometry, single-cell analysis or cytokine profiling when possible, because immune-cell survival, T-cell sequestration or exhaustion, myeloid IL-10 signaling and on-chip immunosuppression can all shape interpretation [78,79,80,81]. Immune-cell-containing organoids are therefore best suited to testing spatial immune attack, antigen escape and combination logic, not to recreating systemic immunity.

### 4.5. ECM, Mechanics and Chip-Based Gradients

Glioma invasion is also shaped by extracellular-matrix composition, stiffness, pore architecture, adhesion and interstitial-like flow. Hydrogel, matrix-engineered and bioprinted systems can tune selected physical constraints; in the evidence cited here, the strongest support concerns stiffness-linked invasion and metabolic adaptation (Table 4). These variables help measure migration mode, invasion distance, invasive-edge morphology and mechanically induced resistance [82]. Because matrix and myeloid cues can also shape treatment escape, ECM-focused experiments should be interpreted alongside tumour-microenvironmental resistance mechanisms rather than as purely physical assays [83].

Mechanical and chip-based models can also improve exposure control. In static culture, nutrient exchange, evaporation, local drug accumulation and diffusion distance vary across wells and batches. Microfluidics does not solve every problem, but it gives investigators control over variables that are otherwise hidden.

Microfluidic systems provide dynamic control of flow rate, shear stress, oxygen gradients, drug gradients and exposure timing; organ-on-chip work in hypoxic GBM further shows how such designs can connect diffusion boundaries to treatment readouts [68]. They are strongest for drug penetration, invasive-boundary analysis and reproducible exposure. Their weaknesses are practical: higher cost, lower throughput and limited comparability across chip geometries or hydrogel formulations. For that reason, chip-based claims should be tied to the controlled variable, not to a broad statement that the model is more physiological.

## 5. Applications of Glioma Organoids in Precision Therapy

**Figure 4:**
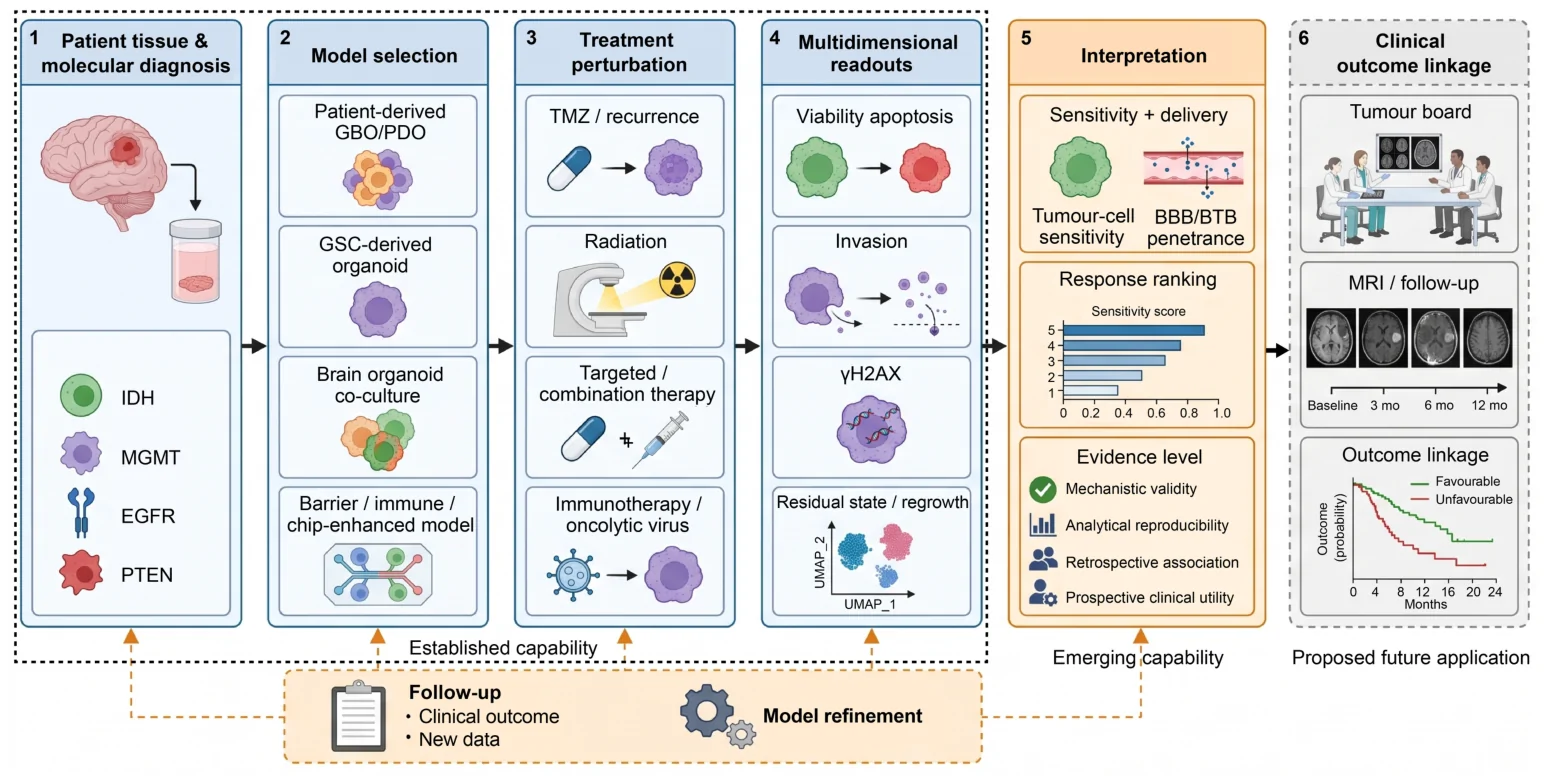
Precision-therapy testing workflow using glioma organoids. A proposed translational framework about organoid assay begins with patient tissue and molecular diagnosis, proceeds through question-matched model selection and treatment perturbation, and ends with multidimensional readouts, response interpretation and clinical linkage. GSC, glioma stem cell; GBO, glioblastoma organoid; PDO, patient-derived organoid; BBB, blood-brain barrier; BTB, blood-tumour barrier; TMZ, temozolomide. Created with BioRender.com.

Glioma precision therapy requires more than target identification (Figure 4). Organoid evidence should be interpreted at four levels: mechanistic validity, analytical reproducibility, retrospective association with patient response, and prospective clinical utility. Most current glioma studies remain at the first two levels, with a smaller number providing retrospective or prospective outcome linkage. Demonstrating differential ex vivo sensitivity does not by itself show that an assay predicts patient benefit or improves outcome. Evidence from gastrointestinal, pancreatic or rectal cancer organoids is therefore indirect support for workflow design rather than validation in glioma [84,85,86,87,88].

### 5.1. Drug Screening and Individualized Prediction

Clinical feasibility requires quantitative reporting. Published proof-of-concept GBO screening has returned candidate options 13–15 days after resection, while short-term organoid protocols commonly require approximately one to three weeks depending on tissue quality, assay complexity and the number of perturbations [32,34,89]. Reports should state the interval from surgery to organoid formation, treatment start and interpretable readout; establishment and assay-failure rates; the proportion of samples evaluable within the therapeutic decision window; and whether clinicians were blinded to organoid results in outcome-linked studies.

The screening design should match both the clinical question and the available material. No universal minimum tissue mass is established, so studies should report wet mass or fragment number, viable tumour-cell fraction, necrotic fraction, sampling region and prior treatment. Fragment-based protocols commonly use viable pieces of approximately 0.5–1 mm, whereas dissociated formats should report input cell number. A focused, replicate-supported panel is preferable to a large library that exhausts material or sacrifices quality control; one proof-of-concept platform tested 41 approved drugs within a clinically relevant interval [89].

Drug screening should distinguish exploratory discovery from clinical-prioritization testing. Exploratory screens may use broader libraries and higher throughput to identify vulnerabilities. Clinical-prioritization assays should focus on plausible brain-penetrant agents and combinations, use clinically achievable unbound exposure where data exist, and include positive, negative and vehicle controls. High-dose in vitro killing should not be described as clinically actionable when BBB/BTB delivery, systemic toxicity or metabolism is not represented.

Patient-derived GBO/PDOs are most useful for drug screening when they preserve the sampled-region architecture and are annotated by integrated diagnosis, treatment history and sampling site. Studies claiming predictive value should report cohort size, establishment rate, assay turnaround, evaluable-sample rate, compounds tested, response definition, replicate performance, concordance with clinical response and whether treatment decisions were influenced prospectively [11,32,34,35,36,37,44,89,90]. Intratumoural sampling bias and reproducibility across fragments, wells, batches and operators should be analyzed rather than treated as technical footnotes.

Core readouts are not interchangeable (Table 5). Sensitivity or resistance should be defined prospectively using dose-response metrics and clinically relevant exposure, with organoid-level rather than only well-level replication. Half maximal inhibitory concentration (IC50), area under the dose-response curve, growth-rate inhibition, apoptosis, clonogenic or post-withdrawal regrowth and the residual resistant-cell fraction answer different questions. Tumour-board integration should present the organoid result alongside molecular evidence, brain exposure, toxicity, trial availability and the level of clinical validation; the assay should support, not override, multidisciplinary judgment.

### 5.2. Temozolomide Resistance and Recurrence Evolution

Post-withdrawal regrowth is especially informative because many therapies shrink organoids at first but leave a viable reservoir. In TMZ assays, resistant cells may localize to hypoxic, slow-cycling or stem-like regions. Tracking those cells after drug removal can show whether treatment has merely delayed growth or has pushed the tumour toward a more resistant state. This readout is closer to the clinical problem of recurrence than a single viability endpoint.

TMZ resistance is well suited to organoid modelling because it emerges under treatment pressure and involves both molecular and microenvironmental mechanisms. MGMT promoter methylation predicts part of the response, but mismatch repair defects, DNA-damage repair, stem-like states, hypoxia and cell-cycle plasticity also contribute [91,92,93,94,95,96,97]. Organoids can model single high-dose exposure, continuous low-dose exposure, pulsed therapy and regrowth after withdrawal; single-cell and PDO studies also support tracking transient resistance states, therapy-driven evolution, autophagy-linked resistance and DNA-damage responses [98,99,100,101].

Useful readouts include MGMT, MMR status, gammaH2AX, RAD51, apoptosis, proliferative recovery, stem-like state enrichment, hypermutation-associated signatures and spatial localization of residual cells. Yet in vitro TMZ pressure cannot fully reproduce the timescale of clinical selection, BBB-limited exposure or the immune and stromal context of recurrence. The best use of organoids is to map residual-cell states and rank rational combinations, not to declare TMZ sensitivity or resistance in isolation.

### 5.3. Radiotherapy Response and Radiosensitization

Radiotherapy studies should report calibrated dosimetry, field geometry, dose rate, total dose, fraction size and schedule, organoid oxygenation and size, timing of concurrent TMZ or radiosensitiser, and post-treatment observation. A single high dose can reveal intrinsic radiosensitivity but does not reproduce repeated repair cycles during clinical fractionation. Fractionated protocols, although slower, can better expose hypoxia-associated survival, delayed DNA-damage repair and post-treatment regrowth.

Radiotherapy response depends on dose, fractionation, oxygenation, DNA-damage repair, cell-cycle state and normal-brain tolerance. Readouts should include γH2AX/53BP1 kinetics, apoptosis, proliferative arrest, clonogenic or post-withdrawal regrowth, invasive-edge behaviour and spatial residual-cell states rather than endpoint viability alone. Paired normal brain organoids or other normal-tissue comparators may help estimate therapeutic index, but they cannot replace clinical dosimetry or neurotoxicity assessment.

Mechanistically, GSC-like cells, N-cadherin-associated adaptation, replication stress and altered metabolism can support radioresistance [102,103,104,105,106]. Clinically relevant organoid experiments should mirror fractionation, TMZ co-treatment and achievable radiosensitizer exposure. Trials combining radiation, TMZ, lomustine or PARP inhibitors show why brain penetrance and toxicity window cannot be ignored [107,108,109,110,111]. Organoids are therefore best used to compare radiosensitization hypotheses and residual-cell behaviour, not to replace dosimetry or clinical toxicity assessment.

### 5.4. Targeted and Combination Therapy

Combination testing should avoid the assumption that more killing in vitro means a better regimen. Sequence, dose intensity and exposure duration can determine whether two agents cooperate or simply add toxicity. Organoids are useful because they can test regrowth after withdrawal and spatial escape, but the result still needs to be interpreted through brain penetrance and tolerability.

Targeted therapy in glioma is difficult because molecular vulnerability, pathway bypass, clonal heterogeneity and BBB penetration often diverge. Organoids allow molecularly annotated testing of EGFR, PI3K/mTOR, CDK, PARP and DNA-repair strategies while retaining spatial heterogeneity [112,113,114,115]. Combination experiments should report dose matrix, sequence, synergy score, clinically achievable concentration, invasion suppression and regrowth after withdrawal. These details matter because apparent in vitro synergy can disappear when brain exposure is inadequate or toxicity is limiting.

Synergy metrics such as Bliss or Loewe can help rank combinations, but they do not prove clinical usefulness unless concentrations are achievable and the schedule is plausible. Organoids are most informative when they show whether a combination suppresses residual-cell regrowth, reduces invasive spread or blocks an adaptive state that a single agent leaves behind.

Brain-penetrant PI3K/mTOR inhibitors, EGFR inhibitors, PARP-inhibitor combinations and other targeted approaches illustrate both opportunity and constraint [112,113,114,115]. The strongest use of organoids is hypothesis-guided testing tied to dominant driver pathways, resistance states and bypass compensation, not broad screening of every available compound. Chip-enabled or BBB/BTB-related systems are preferred when delivery is part of the therapeutic question.

### 5.5. Antiangiogenic Therapy and Tumour-Treating Fields

Antiangiogenic therapy can be interrogated only partially in organoid systems. Vascular-associated or perfused chip models can measure VEGF-pathway perturbation, endothelial organization, permeability, perfusion and spatial drug response, but a tumour-only submerged organoid cannot reproduce systemic vascular normalization, edema control, blood pressure, wound healing or immune-vascular interactions. Bevacizumab-related organoid results should therefore be described as local tumour/vascular responses rather than complete clinical-response models [116].

Tumour-treating fields (TTFields) can be applied to glioma spheroids, organoids, tumour slices and paired normal brain organoids, but experiments must report frequency, field strength, duty cycle, electrode geometry, exposure duration, thermal controls and spatial field distribution [117,118]. These models can examine direct antiproliferative effects, apoptosis and patient-to-patient variability; they do not reproduce scalp/skull field attenuation, device adherence, whole-brain distribution or the complete clinical context of maintenance therapy. TTFields should therefore be treated as a modality-specific preclinical application rather than an automatically patient-predictive assay.

### 5.6. Immunotherapy, Cell Therapy and Oncolytic Viruses

For cell therapy, spatial pattern can be as revealing as total killing. Native-immune-retaining GBOs, autologous reconstructed systems and allogeneic co-cultures should be reported separately. Some CAR-T cells may remain at the surface; others may enter but fail to clear hypoxic or antigen-low regions. Repeated dosing can reveal antigen loss, exhaustion or resistant-state selection, provided HLA compatibility, activation state, tumour-to-effector ratio, medium, viability and exposure duration are documented.

Oncolytic-virus assays also need spatial interpretation. A virus may infect the outer rim but fail to penetrate dense or necrotic regions, or it may spread well but trigger immune clearance in more complex systems. Viral entry, replication, lysis, cytokine release and residual-cell regrowth should therefore be read together. This makes organoids useful for studying how architecture and immune context shape viral therapy.

Immunotherapy requires models that measure spatial killing, immune retention, cytokine state and antigen escape rather than tumour-cell viability alone. Checkpoint blockade has produced limited benefit in unselected recurrent GBM, although neoadjuvant studies suggest that immune state and timing may matter [116,119,120,121,122]. Local organoid systems cannot recapitulate systemic immunosuppression, lymphoid-organ priming, bone-marrow T-cell sequestration, corticosteroid exposure or treatment-related neurotoxicity and systemic toxicity. These limitations should accompany any claim concerning checkpoint blockade or cellular therapy.

CAR-T and related cell therapies fit organoid testing because antigen expression, infiltration depth, repeated killing and residual-cell regrowth can be directly observed. Clinical studies targeting IL13Ralpha2, EGFRvIII, HER2-related or multi-antigen approaches show both activity and escape, including antigen loss and adaptive resistance [123,124,125,126]. Organoid assays should record antigen density, effector:tumour ratio, cytokine profile, killing coverage, antigen-negative survivors and post-treatment regrowth. Donor-matched organoid and tumour-immune organoid studies also underline why efficacy readouts should be paired with off-tumour toxicity or immune-microenvironment context when claims move toward translation [127,128].

Oncolytic viruses add another layer because viral entry, replication, spread, lysis and immune activation all have to be considered. Trials of DNX-2401, recombinant poliovirus, G47Delta and virus-checkpoint combinations show translational potential, while also highlighting delivery route, antiviral immunity and safety constraints [129,130,131,132]. GBOs can measure infection range, replication kinetics, penetration into hypoxic or necrotic regions and residual-cell survival. Immune-cell-containing organoids are preferable when immune activation or immune-mediated viral clearance is central.

### 5.7. Translational Interpretation

Across drugs, radiation, antiangiogenic therapy, TTFields, immune therapy and oncolytic viruses, organoid response should be read as functional evidence rather than a clinical decision by itself. Biological activity, analytical validity, retrospective clinical association and prospective clinical utility should be stated separately. The most useful reports combine model type, molecular annotation, realistic exposure, multidimensional endpoints, normal-brain/barrier context where relevant, assay failure and follow-up linkage. With those elements, organoids may rank or deprioritize regimens and explain resistance; without them, they remain preclinical perturbation assays (Table 6).

## 6. Multi-Omic Integration and Model Quality Control

### 6.1. Multi-Omic Fidelity Validation

**Figure 5:**
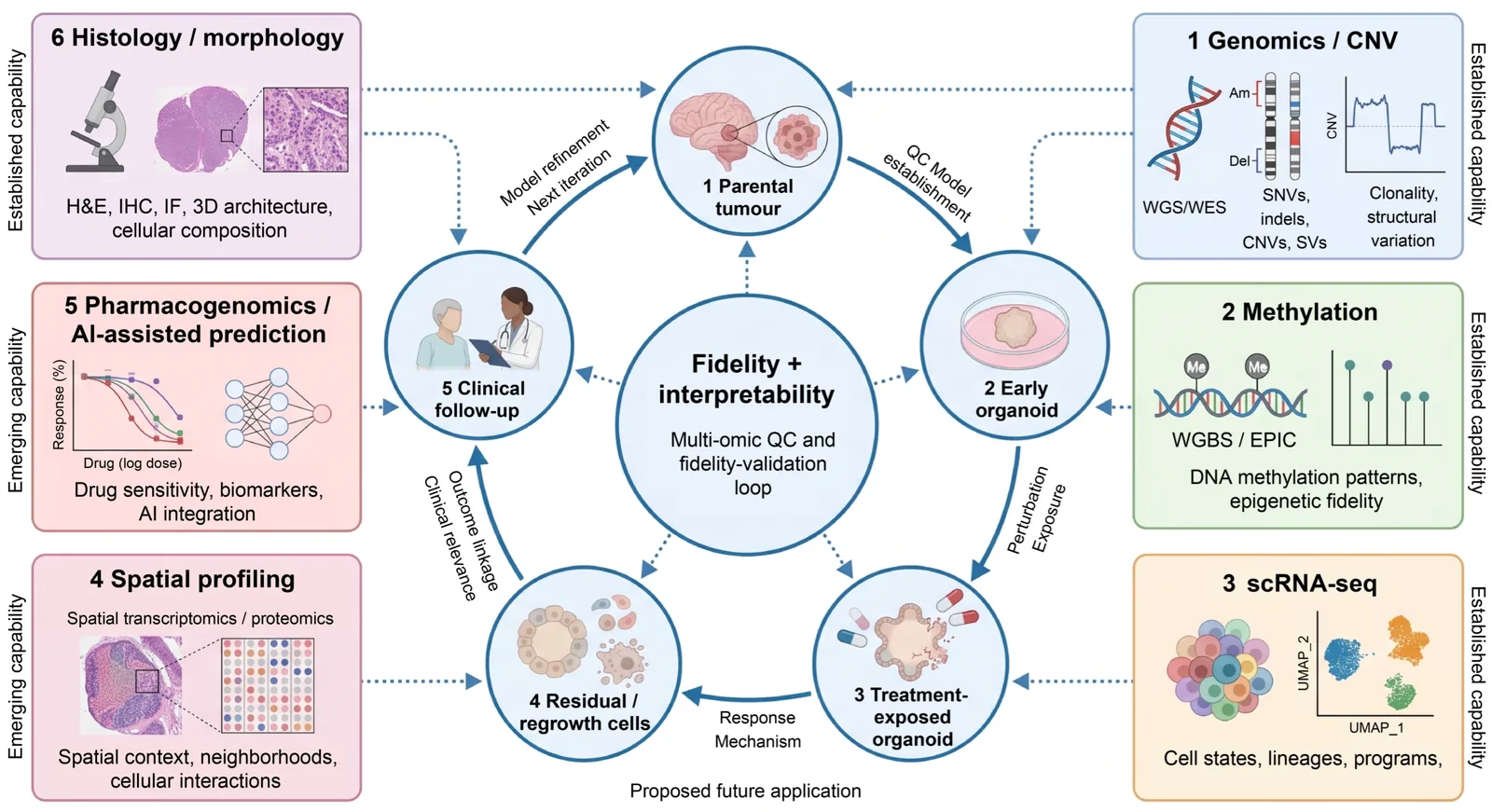
Multi-omic quality control and fidelity validation loop. Parental tumour, early organoid, treatment-exposed organoid, residual or regrowth cells and clinical follow-up are connected through histology, genomics, methylation, single-cell, spatial and pharmacogenomic validation layers. The required layer should be matched to the claim, and analytical validation should precede AI-assisted prediction. WGS, whole genome sequencing; WES, whole exome sequencing; Am, amplification; Del, deletion; WGBS, whole genome bisulfite sequencing; EPIC, methylation microarray. Created with BioRender.com.

Multi-omic validation should be targeted rather than exhaustive. Not every study needs single-cell sequencing, spatial transcriptomics, methylation profiling and proteomics, but every study should justify the fidelity layer it claims (Figure 5). A drug-screening paper may need molecular anchors and response correlation; an invasion paper may need spatial or single-cell readouts at the invasive edge; an engineered model may need evidence that the introduced driver has produced the intended lineage or state. A smaller, well-matched validation panel is often more convincing than a broad but unfocused survey.

Multi-omic validation is needed because morphology alone cannot prove fidelity. Single-cell RNA sequencing can test whether malignant cell states, non-malignant compartments and treatment-induced residual states are retained. Spatial transcriptomics and multiplex imaging can ask whether those states are organized in meaningful niches. General organoid spatial-profiling and live-imaging methods may support this task, but only after adaptation and validation in glioma-specific material [133,134]. Genomics, copy-number analysis and methylation profiling then check whether the organoid still belongs to the same molecular disease class as the parental tumour [135,136,137].

The choice of omic layer should follow the model claim. Whole-genome or copy-number analysis matters when the claim concerns driver preservation or clonal evolution. DNA methylation profiling matters when diagnostic class is central. Single-cell RNA sequencing is useful when the claim concerns cell states, while spatial assays are needed when the argument depends on niche organization. This matching keeps multi-omics from becoming decorative.

For patient-derived organoids, the central question is whether the model preserves the parental tumour well enough for the intended use. Early-passage cultures may retain the original state better but offer fewer experiments; long-term models enable repeated testing but may drift. Pediatric brain tumour organoids and recurrent GBM organoid-guided studies show the value of matching molecular fidelity with treatment history and response data [89,138,139,140]. A small number of well-matched parental tumour-organoid pairs may be more informative than a larger dataset with weak sampling metadata or unclear culture histories.

### 6.2. Pharmacogenomics and AI-Assisted Prediction

Artificial intelligence (AI) is most useful when it connects data types that are otherwise difficult to interpret together. Imaging can measure growth and invasion; sequencing can identify cell states and resistance pathways; clinical records provide outcome and treatment context. A model that integrates these layers may reveal response patterns that no single assay can show. Still, training data must be transparent, and the validation set must be separate enough to avoid optimistic predictions.

Organoid response data become more useful when they are combined with genomic, transcriptomic, spatial and clinical variables. Across cancer types, patient-derived organoid studies have shown that ex vivo sensitivity can complement molecular markers, and glioma-focused work is beginning to test this logic under brain-specific constraints [84,85,86,141,142,143]. For glioma, the integration problem is sharper because response depends on drug penetration, hypoxia, stem-like states, DNA repair and the invasive edge.

AI should be framed as an integration layer, not as a substitute for experimental validation. Models trained on organoid data remain vulnerable to small cohorts, platform bias, batch effects, leakage and incomplete outcome annotation. Broader organoid precision-medicine reviews make the same point: prediction is useful only when assay context and outcome linkage are clear [87,88]. Transfer learning and organoid-guided prediction frameworks suggest that AI may help connect ex vivo response, multi-omic state and clinical outcome when training and validation sets are transparent [144]. In a cautious workflow, AI ranks hypotheses and response patterns; experiments and follow-up determine whether those patterns are real.

### 6.3. Minimum Quality-Control (QC) Reporting

QC should be tiered into mandatory minimum, application-specific and advanced/exploratory layers. Universal numerical cut-offs are not yet justified across all platforms, but every study should pre-specify operational acceptance criteria: sample identity, viable organoid number, size range, mycoplasma status, replicate number, control separation, allowable variability, fidelity anchors and assay evaluability. Failed cultures and excluded wells should be reported with reasons [145,146,147,148].

Mandatory QC establishes that the correct tumour was cultured and that the assay performed reproducibly. Application-specific QC tests the claimed function—for example, permeability and transporter activity for BBB/BTB models, immune retention and HLA/source compatibility for immune models, calibrated dosimetry for radiotherapy, or field/thermal mapping for TTFields. Advanced QC adds single-cell, spatial, methylation, proteomic, inter-laboratory or prospective outcome validation when required by the claim.

At least three independent organoid-level replicates per condition should be reported when material permits, together with replicate variability and a pre-specified rule for non-evaluable assays. Major diagnostic drivers, copy-number profile or methylation class should be concordant with the matched sampled tissue to the extent required by the claim; discordant findings should not be hidden. Positive/negative controls, reference materials, batch effects, operator effects and inter-laboratory benchmarking should be included where applicable (Table 7).

## 7. Standardization, Translational Bottlenecks and Future Directions

### 7.1. Minimum Reporting, Reproducibility and Technical Bottlenecks

Reproducibility begins before culture starts. Anatomical sampling site, primary versus recurrent status, prior therapy, cold-ischemia interval, tissue handling, viable tumour fraction and protocol choice influence later results. Failed cultures should be reported because establishment success and selection bias are part of biological and clinical feasibility, not technical footnotes. Minimum reporting should include the variables in Table 7 and the principal inference the model cannot support.

The next challenge is to move from feasibility studies to comparable evidence. Reproducibility does not require every laboratory to build an identical organoid, but it requires common metadata, explicit acceptance criteria, harmonised response definitions, reference controls and inter-laboratory benchmarking. Complexity should be added only when the new component produces a measurable function and an interpretable gain over a simpler comparator.

The greatest bottleneck is the balance between complexity and interpretability. Adding vessels, immune cells, neural activity, ECM and perfusion can improve a selected function while introducing new sources of variance. Vascular-associated models may lack mature perfusion or barrier function; immune cells may lose viability or shift activation state; brain organoids vary in maturation and regional identity; and chip geometry or hydrogel composition can dominate the readout [149,150,151,152]. A restrained model that measures one niche feature well is preferable to a complex model whose causal variables are unclear.

Major bottlenecks remain in ecosystem reconstruction. Vascularized models often show vessel-like structures but incomplete perfusion and barrier function. Immune-cell-containing models may lose immune-cell viability or shift activation states in culture. Brain organoids vary in maturation, regional identity and neural-network activity, as shown by studies of cortical organoid cell diversity and network development [149,150,151,152]. ECM and chip-based systems improve physical control but reduce throughput and increase technical variability. These issues do not make organoids unreliable; they define the conditions under which a claim should be made.

### 7.2. Clinical Translation and Governance

Ethical governance should follow the same pragmatic logic. Patient-derived organoids are linked to identifiable clinical histories, genomic data and possible treatment decisions, and this makes ethics and governance more than a final administrative step [153,154]. Consent language should cover biobanking, recontact, data sharing, commercial use and whether results may be returned. Patients also need protection from exaggerated claims: an organoid response can inform a discussion, but it should not be presented as a guaranteed prediction of clinical benefit.

Prospective validation should define what counts as success. An organoid assay may help by identifying an ineffective drug, ranking two plausible regimens, explaining recurrence or supporting trial selection. These are different endpoints, and they require different study designs. Reports should state culture success rate, turnaround time, assay failure, discordant cases and response correlation. Organoid results should complement physician judgment and molecular tumour-board evidence rather than override them.

### 7.3. Future Directions

Digital-twin language should be used sparingly. At present, most glioma organoid platforms do not yet integrate imaging, pathology, genomics, longitudinal therapy exposure and clinical outcome at a scale sufficient for autonomous prediction. A more realistic near-term goal is a cross-platform evidence chain in which organoids provide functional perturbation data that can be combined with MRI, spatial pathology and molecular profiling. That step could support digital decision-making without overstating current maturity.

Future glioma organoid research will likely develop along three paths. First, functional precision therapy will move from single-agent viability to regimen-ranking pipelines that include drug penetration, invasion, residual-cell state and regrowth. Second, precision radiotherapy will use organoids and paired normal brain models to explore radiosensitization, DNA-damage repair and therapeutic index [155]. Third, cross-platform evidence chains will combine PDO/GBOs, GSC organoids, co-cultures, BBB chips, PDX models and clinical follow-up according to the question.

**Figure 6:**
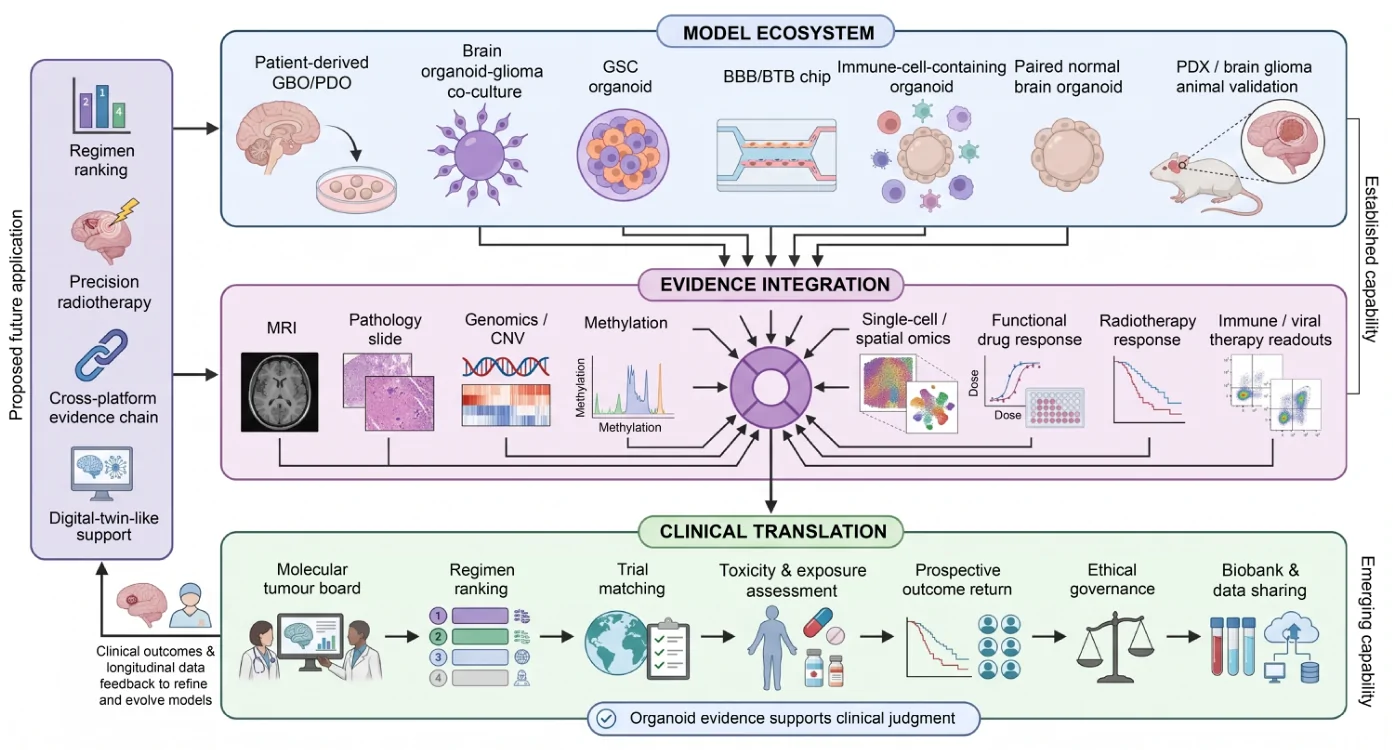
Future translational ecosystem for glioma organoid platforms. Future platforms should integrate model ecosystems, imaging, pathology, multi-omics, functional response, prospective outcome return, governance and biobank data while keeping organoid evidence supportive rather than deterministic. GSC, glioma stem cell; GBO, glioblastoma organoid; PDO, patient-derived organoid. Created with BioRender.com.

Clinical imaging is likely to become part of this evidence chain. MRI defines enhancing tumour, edema, necrosis and recurrence patterns, whereas organoids provide cellular and functional response data. Linking organoid-derived sensitivity maps with imaging, spatial omics and clinical outcomes may support future digital-twin-like frameworks (Figure 6). For now, this should remain a cautious direction rather than a claim of clinical readiness [156,157].

The next phase of the field will probably be less about inventing new organoid variants and more about making existing variants comparable. Shared terminology, minimal metadata, common response metrics and transparent reporting of failed cultures will matter as much as technical innovation. Without these elements, even sophisticated models will remain difficult to compare across centers.

Standardization should not force every laboratory into one identical model. Instead, it should make the model–question relationship explicit and the evidence chain auditable. The field needs fewer demonstrations that organoids can be built and more studies showing when organoids change interpretation, explain resistance or improve regimen prioritization. That shift will define the next stage of glioma organoid research.

## 8. Conclusions

Taken together, glioma organoids are most convincing when investigators neither oversell them as miniature patients nor reduce them to convenient screening tools. Their real strength lies in the middle ground: they make patient-derived biology experimentally accessible while forcing researchers to specify what has, and has not, been reconstructed.

Glioma organoids have changed how experimental neuro-oncology can connect patient tissue, microenvironmental biology and therapeutic testing. Their value does not lie in replacing two-dimensional models, animal models or clinical trials. It lies in adding a patient-derived functional layer that can preserve aspects of tumour architecture, model selected niche pressures and test therapeutic perturbations under controlled conditions.

Their most productive use remains question-driven. Patient-derived GBO/PDOs are strongest for sampled-region fidelity and patient-specific perturbation; GSC-derived organoids for reproducible cell-intrinsic mechanisms; brain organoid–glioma co-cultures for invasion and neural context; engineered brain organoids for driver causality; and vascular-associated, immune-cell-containing or chip-based platforms for selected perfusion, immune or delivery questions. Acute slices and animal models remain essential complementary comparators.

The field should now move from model demonstration toward validated decision support. That transition will require transparent reporting, multidimensional QC, clinically realistic exposure, multi-omic and spatial validation, prospective outcome linkage and ethical governance. If these standards are met, glioma organoids may become a practical bridge among mechanism, functional precision medicine and clinical interpretation. If they are not, they will remain elegant models with uncertain clinical weight. The distinction is important for a young field: predictive value must be earned through carefully designed validation, not assumed from model complexity.

## Figures and Tables

**Table 1:** Practical boundaries among common three-dimensional glioma models.

Term	Core Meaning	Typical Use in Glioma Research
Spheroid	Three-dimensional aggregate formed by cell clustering	Geometry and basic drug response; limited evidence of tissue hierarchy
Neurosphere	Sphere enriched for neural or glioma stem-like cells	Stemness, clonogenicity, invasion and resistance mechanisms
Tumoroid	Broad tumour-derived three-dimensional structure	Useful descriptor when standards for organoid fidelity are not fully met
Organoid	Self-organizing three-dimensional tissue model with partial architecture and function	Mechanism, niche modelling and treatment perturbation with defined validation
GBO/PDO	Patient-derived glioblastoma or glioma organoid	Parental tumour fidelity, individualized response testing and outcome-linked studies

**Table 2:** Scope of organoid modelling across major molecularly defined glioma entities.

Molecular Entity	Representative Organoid Approaches	Best-Supported Questions	Key Establishment or Fidelity Challenge	Current Evidence Boundary
Glioblastoma, IDH-wildtype	Fragment-based GBO/PDO; GSC-derived organoid; brain co-culture; vascular/immune/chip systems	Heterogeneity, invasion, treatment response, residual states	Regional sampling, necrosis, treatment history, clonal/state drift	Largest evidence base; clinical utility remains incompletely validated
Astrocytoma, IDH-mutant	Lower-grade patient-derived organoids; engineered organoids	IDH biology, progression, treatment-induced evolution	Slow growth; possible loss of IDH-associated metabolic and epigenetic state	Limited cohorts and outcome linkage
Oligodendroglioma, IDH-mutant and 1p/19q-codeleted	Patient-derived lower-grade organoids; selected engineered systems	Lineage fidelity, codeletion-associated biology, therapy response	Low establishment rate in some settings; culture selection and lineage drift	Sparse direct evidence
Diffuse midline glioma, H3K27-altered, and other paediatric diffuse high-grade gliomas	Region-specific engineered organoids; patient-derived/immune co-cultures	Developmental context, invasion, antigen-directed therapy	Limited tissue, age/region specificity, incomplete maturation	Emerging mechanistic and preclinical evidence
Recurrent or treated gliomas and other entities	Short-term patient-derived organoids/explants	Therapy-selected states, recurrence-specific screening	Prior therapy, low viable-cell fraction, sampling bias	Predominantly proof-of-concept or retrospective evidence

**Table 3:** Question-driven comparison of organoid and complementary glioma models.

Model	Typical Timing/Throughput/Relative Cost	Features Preferentially Retained	Best-Suited Applications	Important Compatibility	Principal Inference Not Supported
2D line or GSC/neurosphere	Days; high throughput; low cost	Cell-intrinsic pathways; expandable clones	Genetic perturbation, mechanism, broad screening	Radiation and repeated drug selection	Native architecture, multicellular niche or patient-level prediction
Acute tumour slice/explant	Hours to days; low-moderate throughput; moderate cost	Local architecture, native stromal/immune cells for a limited interval	Short-term pharmacology, spatial response	Radiation, imaging, local immune studies	Long-term evolution, expansion or systemic delivery
Patient-derived GBO/PDO	Approximately 1–3 weeks for short-term assays; moderate throughput/cost	Sampled-region architecture, heterogeneity, selected native components	Patient-specific perturbation, residual states, outcome-linked studies	Drug, radiation, imaging, selected immune assays	Whole-tumour representation, systemic pharmacology or proven clinical benefit
GSC-derived organoid	Days to weeks; moderate-high throughput; moderate cost	Expandable stem-like and resistant states	Mechanistic perturbation, rescue, repeated selection	Drug, radiation, longitudinal imaging	Complete patient ecosystem or regional heterogeneity
Brain organoid–glioma co-culture/assembloid	Weeks to months including host organoid generation; low throughput; high cost	Human neural context and tumour–brain interface	Invasion, neural interaction, host-dependent state change	Live imaging, electrophysiology, selected drug studies	Mature adult brain, complete vasculature/immunity or patient response
Engineered brain tumour organoid	Weeks to months; low and moderate throughput; high cost	Defined developmental context and driver combinations	Tumour initiation, driver cooperation, lineage effects	CRISPR, imaging, developmental studies	Authentic patient evolution or treatment history
Vascular/immune/chip-enhanced organoid	Weeks; low throughput; high cost and standardization burden	Selected barrier, immune or gradient variable	Delivery, perfusion, immune killing, spatial exposure	Permeability, cell therapy, nanoparticles	Whole-body PK, systemic immunity, toxicity or automatic clinical fidelity
Orthotopic PDX/GEMM	Months; low throughput; very high cost	In vivo vascular, organ and systemic context	Tumour growth, exposure, toxicity, organism-level validation	Surgery, radiation, systemic therapy	Rapid patient-specific decision support; species-independent human immunity

**Table 4:** Microenvironmental elements, functional validation and artefact controls.

TME Component	Model	Required Functional Validation	Essential Artefact/Control Strategy	Therapeutic Interpretation
Hypoxia/necrosis	Patient-derived GBO; spheroid; GBM-on-chip	Oxygen/hypoxia mapping, metabolism, spatial proliferation and treatment response	Pre-specified size range; size-matched controls; controlled oxygen/perfusion perturbation; matched tissue	Distinguishes biological hypoxic niches from diffusion-limited culture stress
Neuron–tumour niche	Brain organoid co-culture; GLICO; assembloid	Invasion metrics plus synaptic markers, calcium imaging or electrophysiology when neural coupling is claimed	Host-only and tumour-only controls; neuronal-activity perturbation; organoid maturation metadata	Separates migration from functional activity-dependent growth or synaptic integration
Vessels and BBB/BTB	Vascular-associated organoid; endothelial co-culture; BBB chip	Permeability, barrier resistance, transporter expression, perfusion and spatial drug tracing	Reference barrier control; flow/no-flow comparison; free-drug exposure; endothelial-marker-only claims avoided	Separates tumour-cell sensitivity from delivery competence
Immune suppression	Native-immune-retaining or reconstructed autologous/allogeneic models	Immune retention, infiltration, cytokines, spatial killing, exhaustion and antigen loss	HLA/source/activation/ratio controls; immune-only and tumour-only controls; duration and viability	Supports local immune hypotheses but not systemic immunity or toxicity
ECM and gradients	Hydrogel, bioprinting, microfluidic perfusion	Stiffness, pore structure, shear stress, diffusion curves, invasion and spatial response	Matrix-only controls; batch characterisation; geometry and flow reporting	Defines mechanical or exposure effects without assuming whole-tissue physiology

**Table 5:** Non-interchangeable endpoints in glioma organoid treatment studies.

Endpoint	What It Measures	Strength	Important Limitation
IC50	Concentration producing 50% effect relative to the fitted baseline	Familiar dose-response summary	Depends on curve range and exposure duration; does not capture regrowth or achievable exposure
Area under the dose-response curve	Integrated response across tested concentrations	Uses the full curve and supports ranking	Sensitive to concentration range and normalization; cross-study comparison requires harmonisation
Growth-rate inhibition	Drug effect adjusted for baseline proliferation	Reduces bias from different growth rates	Requires reliable longitudinal measurements and model assumptions
Apoptosis/DNA-damage endpoint	Mechanistic cell-death or damage response	Clarifies pharmacodynamic mechanism	Early response may not predict durable control
Clonogenic or post-withdrawal regrowth	Capacity of residual cells to resume growth	Closer to recurrence and durable response	Longer assay; affected by culture selection and endpoint definition
Residual resistant-cell fraction	Proportion and state of surviving cells after treatment	Captures spatial and cellular escape	Requires single-cell/spatial or robust imaging analysis; threshold must be pre-specified

**Table 6:** Precision-therapy applications, evidence level and interpretation criteria.

Application	Preferred Model	Core Readouts	Minimum Evidence for Interpretation	Principal Boundary
Drug screening	Patient-derived GBO/PDO; barrier/chip model when delivery matters	Dose-response metric, apoptosis, invasion, regrowth, residual states	Analytical reproducibility plus achievable unbound exposure; outcome linkage for predictive claims	Systemic PK, metabolism, toxicity and BBB/BTB may be absent
TMZ resistance	GBO/PDO; GSC-derived organoid; pulsed selection model	MGMT/MMR, DNA damage, residual states, regrowth	Mechanistic validity; retrospective/prospective outcome correlation for prediction	Clinical selection timescale and systemic context incompletely reproduced
Radiotherapy	GBO; co-culture; paired normal organoid	Calibrated dosimetry, γH2AX/53BP1, hypoxia, invasion, regrowth	Reproducible fractionation and normal-tissue comparator	Cannot replace clinical dosimetry or neurotoxicity assessment
Targeted/combination therapy	Molecularly annotated organoid; BBB/chip model	Sequence, synergy, free exposure, residual cells	Mechanistic and analytical validity; brain exposure and toxicity evidence	In vitro synergy does not demonstrate patient benefit
Antiangiogenic therapy	Vascular-associated/perfused model	Perfusion, permeability, endothelial response, spatial tumour effect	Functional vascular readouts and matched controls	Systemic vascular normalization, edema and toxicity are not reproduced
TTFields	Organoid/spheroid/slice with calibrated field; paired normal organoid	Proliferation, apoptosis, regrowth, spatial field-response	Frequency/intensity/duty-cycle/thermal validation	Skull/scalp distribution, adherence and whole-brain context absent
Cell therapy/oncolytic virus	Native-immune-retaining or autologous reconstructed GBO; dynamic co-culture	Infiltration, killing, cytokines, antigen loss, viral spread	Immune-source/HLA/ratio controls; prospective linkage for clinical utility	Systemic immunity, neurotoxicity and delivery remain difficult

**Table 7:** Tiered QC, operational acceptance criteria and reporting requirements.

Tier/Domain	Required Information or Assay	Operational Acceptance Principle	Reporting Requirement
Mandatory: sample metadata	Diagnosis, sampled region, primary/recurrent status, prior therapy, processing interval, viable/necrotic fraction	Sample identity and eligibility defined before culture	Report all attempted samples and reasons for failure/exclusion
Mandatory: culture quality	Starting material, matrix/support, size distribution, timepoint/passage, viable organoid number, mycoplasma test	Pre-specified evaluable-size/viability range; contamination negative	Report establishment rate, attrition and batch/operator effects
Mandatory: molecular identity	At least one diagnosis-linked driver plus pathology; CNV/methylation when central to the claim	Concordance with matched sampled tissue; discordance investigated	Clarify whether fidelity refers to fragment, region or whole specimen
Mandatory: assay validity	Dose/exposure, duration, controls, organoid-level replicates, response definition and statistics	Pre-specified replicate number, control performance and non-evaluable rule	Report raw distribution/variability, excluded wells and assay-failure rate
Application-specific: BBB/BTB	Permeability or resistance, transporters, perfusion and spatial drug mapping	Functional barrier performance against a reference control	Do not infer function from endothelial markers alone
Application-specific: immune	Cell source, autologous/allogeneic status, HLA, activation, ratio, retention, viability	Pre-specified immune viability/retention and source-matched controls	Report medium, duration, cytokines and residual-cell mapping
Application-specific: radiation/TTFields	Dosimetry/fractionation/oxygenation or frequency/intensity/duty cycle/thermal mapping	Calibrated physical exposure with sham- and normal-tissue controls	Report equipment geometry and post-treatment regrowth
Advanced/exploratory	Single-cell/spatial/methylation/proteomic fidelity; inter-laboratory benchmark; clinical linkage	Validation layer matched to the strength of the claim	Report reference controls, batch correction, blinded analysis and discordant outcomes

## Data Availability

No new data were created or analyzed in this study. Data sharing is not applicable to this article.

## Abbreviations

The following abbreviations are used in this manuscript:

BBB	blood–brain barrier
BTB	blood–tumour barrier
CA9	carbonic anhydrase 9
CAR-T	chimeric antigen receptor T cell
CNV	copy number variation
ECM	extracellular matrix
GBM	glioblastoma
GBO	glioblastoma organoid
GSC	glioma stem cell
HIF	hypoxia-inducible factor
IC50	half-maximal inhibitory concentration
IDH	isocitrate dehydrogenase
MGMT	O6-methylguanine-DNA methyltransferase
MDSC	myeloid-derived suppressor cell
MMR	mismatch repair
PDO	patient-derived organoid
PARP	poly ADP ribose polymerase
TAM	tumour-associated macrophage
TMZ	temozolomide
TME	tumour microenvironment
WES	whole exome sequencing
WGS	whole genome sequencing
WGBS	whole genome bisulfite sequencing
